# Dietary and lifestyle inflammatory scores are associated with increased risk of metabolic syndrome in Iranian adults

**DOI:** 10.1186/s13098-021-00648-1

**Published:** 2021-03-12

**Authors:** Hossein Farhadnejad, Karim Parastouei, Hosein Rostami, Parvin Mirmiran, Fereidoun Azizi

**Affiliations:** 1 Health Research Center, Life Style Institute, Baqiyatallah University of Medical Sciences, Tehran, Iran; 2 Nutrition and Endocrine Research Center, Research Institute for Endocrine Sciences, Shahid Beheshti University of Medical Sciences, Tehran, Iran; 3 Department of Clinical Nutrition and Dietetics, Faculty of Nutrition Sciences and Food Technology, National Nutrition and Food Technology Research Institute, Shahid Beheshti University of Medical Sciences, Tehran, Iran; 4 Endocrine Research Center, Research Institute for Endocrine Sciences, Shahid Beheshti University of Medical Sciences, Tehran, Iran

**Keywords:** Diet, Lifestyle, Inflammation, Metabolic syndrome, MetS, Adult

## Abstract

**Background:**

In the current study, we aimed to investigate the association of dietary inflammation scores (DIS) and lifestyle inflammation scores (LIS) with the risk of metabolic syndrome (MetS) in a prospective population-based study.

**Methods:**

A total of 1625 participants without MetS were recruited from among participants of the Tehran Lipid and Glucose Study(2006–2008) and followed a mean of 6.1 years. Dietary data of subjects were collected using a food frequency questionnaire at baseline to determine LIS and DIS. Multivariable logistic regression models, were used to calculate the odds ratio (ORs) and 95 % confidence interval (CI) of MetS across tertiles of DIS and LIS.

**Results:**

Mean ± SD age of individuals (45.8 % men) was 37.5 ± 13.4 years. Median (25–75 interquartile range) DIS and LIS for all participants was 0.80 (− 2.94, 3.64) and 0.48 (− 0.18, − 0.89), respectively. During the study follow-up, 291 (17.9 %) new cases of MetS were identified. Based on the age and sex-adjusted model, a positive association was found between LIS (OR = 7.56; 95% CI 5.10–11.22, P for trend < 0.001) and risk of MetS, however, the association of DIS and risk of MetS development was not statistically significant (OR = 1.30;95% CI 0.93–1.80, P for trend = 0.127). In the multivariable model, after adjustment for confounding variables, including age, sex, body mass index, physical activity, smoking, and energy intake, the risk of MetS is increased across tertiles of DIS (OR = 1.59; 95% CI 1.09–2.33, P for trend = 0.015) and LIS(OR = 8.38; 95% CI 5.51–12.7, P for trend < 0.001).

**Conclusions:**

The findings of the current study showed that greater adherence to LIS and DIS, determined to indicate the inflammatory potential of diet and lifestyle, are associated with increased the risk of MetS.

## Background

Metabolic syndrome (MetS) is a clustering of various cardiometabolic risk factors that includes central obesity, hypertriglyceridemia, low high-density lipoprotein cholesterol (HDL-C), elevated blood pressure, and hyperglycemia [1], which is strongly related to an increased risk of developing type 2 diabetes, cardiovascular disease (CVD), cancers, and all-cause mortality [2, 3]. This global epidemic abnormality can be the main cause of morbidity and mortality not only in the developed world but also in developing countries [4]. Metabolic syndrome is considered a growing public health problem because of its high global prevalence, which has affected 20%–25% of adults worldwide [4], 25% in the United States [5], and 30% in Iran [6].

The pathogenesis of MetS is complex and remains to be fully identified. However, insulin resistance, dysregulation of lipid metabolism, and the development of a state of chronic inflammation playing important role in the pathogenesis of MetS, which is a complex pathophysiology [3, 7]. Genetic predisposition and lifestyle factors play a major role in the pathogenesis of MetS [8, 9]. The main causative lifestyle risk factors of MetS, include smoking, physical inactivity, alcohol consumption, and especially inappropriate dietary intakes [10, 11]. Also, chronic inflammation, characterized by higher levels of inflammatory factors such as tumor necrosis factor-α, C-reactive protein, and interleukins, is a well-known risk factor related to the development of MetS [7]. MetS are considered to be a pro-inflammatory state is mostly caused by unhealthy lifestyle and inappropriate dietary pattern; because unhealthy lifestyle factors including unhealthy diet, central obesity, physical inactivity, and cigarette smoking collectively play an important role in the prediction of systemic inflammation [12, 13].

Dietary inflammatory index (DII) as a pre-defined dietary inflammation score was developed to investigate the contributions of dietary exposures on the inflammatory status and consequently the risk of chronic diseases such as MetS and cardiovascular diseases (CVDs) [14]. However, DII mostly includes specific nutrients without considering the nutrient interactions in body homeostasis and other effects of unmeasured and unknown anti/pro-inflammatory compounds of whole foods and beverages. Recently, potential pro or anti-inflammatory effect of lifestyle and dietary pattern has been determined by novel inflammatory indices, including dietary inflammation scores (DIS) and lifestyle inflammation scores (LIS) in the Byrd et al. study to assess the collective contributions of lifestyle and diet exposures to systemic inflammation [15]. Some studies have been reported that higher score of LIS and DIS is associated with increased risk of chronic diseases and its mortality such as cancers, all-cause mortality, and cancer- and cardiovascular disease-specific mortality [16–18], however, to the best of our knowledge, no study has yet investigated the relationship between DIS and LIS and risk of MetS.

The present study aimed to investigate the associations between DIS and LIS and the risk of MetS among Iranian adult participants.

## Methods

### Study participants

The current study was performed in the framework of the Tehran Lipid and Glucose Study (TLGS), a population-based cohort study conducted to investigate the risk factors of chronic diseases among a representative urban population of Tehran, including 15 005 participants aged ≥ 3 years [19]. The first survey of TLGS (a cross-sectional study) is initiated in March 1999 and data collection, conducted prospectively at 3 years intervals, is ongoing; the details of the TLGS have been explained previously [19].

**Fig. 1:**
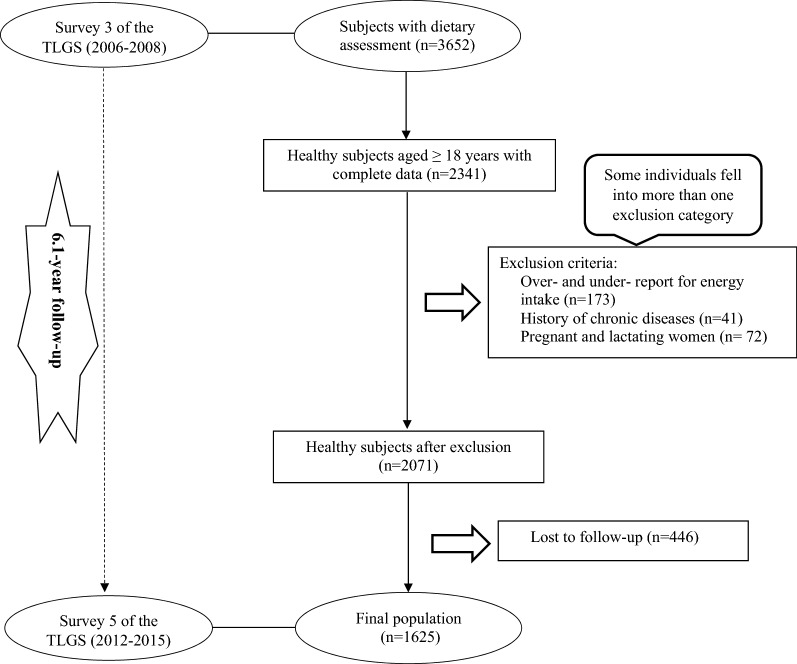
Flow chart of the Tehran Lipid and Glucose Study (TLGS) participants

In the third survey of the TLGS (2006–2008), of 12,523 participants, dietary data of 3652 randomly selected subjects have been determined. For the present study, 2341 adult populations (aged > 18 years) with complete baseline data and free of MetS at baseline were selected. After excluding participants who under-reported or over-reported energy intakes (< 800 kcal/d or > 4200 kcal/d, respectively) (n = 140), or were on specific diets for hypertension, diabetes, or dyslipidemia (n = 33), those with a history of myocardial infarction, cerebral vascular accident, cancers (n = 41), and pregnant and lactating women (n = 72); 2071 participants were followed until March 2015, for a mean period of 6.1 years from the baseline phase; some individuals fell into more than one exclusion category. Finally, after excluding the participants who left the study (n = 446), final analyses were conducted on data of 1625 adults (Fig. 1). It should be noted, the sample size was also calculated using the G power software based on the Shakeri et al. study, which has reported that higher adherence to the empirical dietary inflammatory pattern respectively was 1.75 (95% CI 1.21–2.54) and 1.43 (95% CI 1.03–1.97) times more likely to result in being MetS and abdominal obesity compared with those with low adherence [20]. The minimum sample size needed for this study was 1320 participants with considering a confidence interval of 95% (α = 0.05), study power (1−β) of 80% with the mean MetS incidence of 17.5% in the Iranian adult population [20] to detect an odds ratio of 1.4 for MetS according to DIS score. Therefore, the final population of TLGS that remained for the final analysis of the current study with considering the inclusion and exclusion criteria was sufficient to analyze the relationship between these dietary indicators and the risk of MetS.

### Dietary assessment

The dietary intakes of individuals over the previous year were assessed using a valid and reliable 168-semi-quantitative food frequency at baseline [21]. Expert nutritionist, with at least 5 years’ experience in TLGS, asked participants to designate their consumption frequency for each food item during the previous year on a daily, weekly, or monthly basis; portion sizes of consumed foods, reported in household measures, were then converted to grams. Since, the Iranian Food Composition Table (FCT) is incomplete and has limited data on the nutrient content of raw foods and beverages, the United States Department of Agriculture (USDA) FCT was used. For national foods not listed in the USDA FCT, the Iranian FCT was used.

The inflammatory scores of participants were determined using dietary data derived from FFQ. The Byrd et al. study recently has defined proposed the DIS and LIS [15]. DIS has 19 components originally but due to lack of data on supplement intakes, we calculated the overall score based on 18 food groups including leafy greens and cruciferous vegetables, legumes, refined grains, and starchy vegetables, apples and berries, deep yellow or orange vegetables and fruit, tomatoes, other fruits, and real fruit juices, other vegetables, added sugars, red and organ meats, processed meats, fish, poultry, high-fat dairy, low-fat dairy and tea, nuts, and other fats. Each food group was standardized and then the values were summed.

Body mass index (BMI), physical activity, and smoking status were used to calculate the LIS score. First, a dummy variable was created from each component, the components multiplied by proposed regression coefficients and then all the weighted values were summed to calculate the LIS.

### Physical activity assessment

A modifiable activity questionnaire (MAQ) was used to determine the physical activity levels of participants, which was previously modified and validated among Iranians [22]. A trained interviewer asked participants to report the activities that they had participated in at least 10 times during the past year in their leisure times and then identified the frequency and duration for each leisure-time physical activities. We summed the total number of minutes per year, which was calculated for every physical activity and then we divided by 60 and 52 to estimate the hours per week of total leisure-time physical activity. Metabolic equivalent hours per week (MET-h/wk) of leisure time activity was computed by multiplying the number of hours per week of each leisure time activity to MET. Also, based on the MAQ questionnaire, participants were asked to identify the number of month and hours participated in physical activity at work (including standing, housework, work activities more intense than standing) over the past 12 months. The assessment of occupational activity was based on using the number of hours per week of light, moderate, and hard intensity activity, summed to report hours per week of occupational activity over the past 12 months. Final occupational (MET-h/wk) activity was determined by multiplying the number of hours per week of each three categories of occupational activity to MET values. We reported the total physical activities of participants MET-h/wk by adding leisure-time physical activity to occupational activity.

### Demographic, anthropometric, and lifestyle measures

Trained interviewers used a standard questionnaire to collect information on demographic data, medical history, medications, and smoking habits at baseline (2006–2008). We defined the smoking status in participants based on World Health Organization guidelines [23]. In the TLGS questionnaire, smoking was classified into yes/no groups; ‘yes’ defined individuals who smoked cigarettes as daily or occasionally or ex-smokers and ‘no’ defined the participants who are non-smokers. A standardized mercury sphygmomanometer with an accuracy of 2 mmHg was used to determine the blood pressure of each participant twice on the right arm with a minimum interval of 30 s via after a 15-min rest sitting on a chair; the mean of the two measurements was considered to be the blood pressure of the participant.

A digital scale was used to measure the weight of participants to the nearest 100 g with minimal clothing and without shoes. Height was measured by a tape meter to the nearest 0.5 cm, in a standing position without shoes. BMI was computed as weight (kg) divided by the square of the height (m^2^). Waist circumference (WC) was measured using an unstretched shape tape meter and recorded to the nearest 0.1 cm. WC measurements were performed at the abdominal level, at the umbilical level, over light clothing, and without any pressure to the body surface.

### Biochemical measurements

A blood sample was taken after 12–14 h of overnight fasting in a sitting position based on the standard protocol. The blood samples were centrifuged within 30–45 min of collection. All blood analyses were performed at the TLGS research laboratory. The Selectra 2 auto-analyzer (Vital Scientific, Spankeren, The Netherlands) was used to analyze the samples. We used an enzymatic colorimetric method with glucose oxidase to determine fasting plasma sugar (FPS). Both inter- and intra-assay coefficient variations were 2.2% for FPS. For the oral glucose tolerance test, 82.5 g of glucose monohydrate solution (equivalent to 75 g anhydrous glucose) was administered orally to subjects, aged > 20 years. A second blood sample was taken 2 h after glucose ingestion. Triglycerides (TGs) level was measured using an enzymatic colorimetric analysis with glycerol phosphate oxidase. Total cholesterol (TC) was measured with cholesterol esterase and cholesterol oxidase, using the enzymatic colorimetric method. High-density lipoprotein cholesterol was measured after precipitation of the apolipoprotein B-containing lipoproteins with phosphotungistic acid. All analyses were performed using commercial kits (Pars Azmoon Inc., Tehran, Iran). Inter-assay and intra-assay coefficients of variations were 1.6% and 0.6% for TGs, 2% and 0.5% for HDL-C, and 2% and 0.5% for TC, respectively. We used the Friedewald formula to determine low-density lipoprotein cholesterol from the serum TC, TG, and HDL-C concentrations.

### Definitions

Metabolic syndrome was determined based on the joint interim statement as the presence of any 3 of 5 following factors [24]: (a) central obesity as WC ≥ 95 cm for both genders, according to the new cutoff points of WC for Iranian Adults [25]; (b) FPS ≥ 100 mg/dl or using anti-diabetic medications; (c) fasting TGs ≥ 150 mg/dl or use of anti-lipid medications; (d) fasting HDL-C < 50 mg/dl for women and < 40 mg/dl for men or drug treatment; and (e) high BP was defined as SBP ≥ 130 mm Hg, DBP ≥ 85 mm Hg, or use of antihypertensive medications.

### Statistical analysis

The Statistical Package for Social Sciences (Version 15.0; SPSS, Chicago, IL) was used to perform all analyses. The normality of the variables was checked using a histogram chart and Kolmogorov–Smirnov test. Chi-square and independent two sample t-tests were used for the comparison of categorical and continuous variables, respectively, between individuals with and without MetS. Baseline characteristics of the individuals are expressed as the mean ± SD or median (25–75 interquartile) for continuous variables and percentages for categorical variables. Participants were also categorized according to tertiles of DIS and LIS cutoff points; linear regression and chi-square analysis were used to test the trends of continuous and categorical variables across tertiles of DIS and LIS. Multivariable logistic regression models were used with MetS as the dependent variable and DIS and LIS as independent variables to estimate the risk of 6.1-year incident outcomes. The odds ratio (ORs) and 95% confidence intervals (CIs) were reported for logistic regression models. The first tertile of DIS and LIS was considered as the reference group. Potential confounders, including sex, age, BMI, physical activity, smoking, daily energy intake were adjusted in multivariable logistic regression models. P-values < 0.05 were considered to be statistically significant. We have also conducted an additional analysis using the area under receiver operating characteristic (ROC) curves analysis to evaluate the abilities of the baseline DIS and LIS to predicting of MetS incident.

## Results

The mean age of participants (45.8% male) was 37.5 ± 13.4 years at baseline. During an average of 6.1 years of follow-up, 291(17.9%) new cases of MetS were identified. The median (25–75 interquartile range) of DIS and LIS for all participants were 0.80 (−  2.94, 3.64) and 0.48 (− 0.18, − 0.89), respectively.

Baseline socio-demographic, biochemical characteristics, dietary intake of the participant based on MetS status are shown in Table 1. Compared with subjects without MetS, participants with MetS were significantly older, high smoked, higher anti-diabetic and antihypertensive medications, and had lower education levels and higher levels of FBS, 2-h postprandial blood sugar, TGs, LDL-C, BMI, WC, SBP, and DBP at baseline (P < 0.05). No significant differences in physical activity level, corticosteroid use, HDL-C, and TGs: HDL-C ratio were observed between participants in the MetS and non-MetS groups. Based on the findings of Table 1, compared with Non-MetS subjects, participants with MetS had a higher intake of energy, total fat, and saturated fatty acids, and lower intakes of dietary fiber and magnesium. However, the intakes of other nutrients did not differ significantly between the two above-mentioned groups.

**Table 1:** Baseline characteristics of participants according to the development of the metabolic syndrome

	MetS status at follow-up		P-value
	MetS (n = 291)	Non-MetS (n = 1334)	
Baseline demographic and biochemical data			
Age (year)	45.5 ± 12.1	35.7 ± 13.0	< 0.001
Male, %	40.0	47.1	0.025
Body mass index (Kg/m^2^)	29.3 ± 4.4	24.9 ± 4.0	< 0.001
Waist circumference (cm)	94.9 ± 10.5	83.8 ± 11.9	< 0.001
Physical Activity (MET.min/wk)	68.4 (31.8–104.2)	72.9 (34.7–107.2)	0.177
Academic education (graduated), n (%)	18.6	27.9	0.001
Employed, (%)	85.6	81.4	0.096
Smoking, (%)	8.7	13.0	0.044
Fasting blood sugar (mg/dl)	91.1 ± 18.2	85.0 ± 8.8	< 0.001
2-h postprandial blood sugar	106.2 ± 22.5	90.0 ± 40.5	< 0.001
Triglycerides (mg/dl)	127.5 ± 62.1	115.4 ± 66.2	0.002
Low density lipoprotein- Cholesterol (mg/dl)	121.4 ± 30.9	109 ± 32.1	< 0.001
High density lipoprotein- Cholesterol (mg/dl)	44.2 ± 9.2	44.0 ± 10.4	0.718
TGs:HDL-C ratio	3.06 ± 1.91	2.92 ± 2.30	0.334
Systolic blood pressure (mmHg)	116.1 ± 15.4	106.4 ± 13.0	< 0.001
Diastolic blood pressure (mmHg)	76.4 ± 9.1	70.2 ± 9.2	< 0.001
Anti-diabetic medications (%)	2.1	0.4	0.013
Anti-lipid medications (%)	3.4	2.3	0.064
Antihypertensive medications (%)	2.7	0.5	0.012
Corticoestroeids medications (%)	2.0	1.7	0.669
Dietary intakes			
Energy (Kcal/d)	2284 ± 708	2191 ± 707	0.043
Carbohydrate (g/day)	314.2 ± 115.8	302.9 ± 112.6	0.131
Protein (g/day)	77.3 ± 26.5	74.3 ± 26.0	0.066
Fat (g/day)	80.0 ± 31.3	75.8 ± 28.3	0.033
Saturated fatty acids (g/1000 kcal)	11.9 ± 6.7	11.3 ± 3.1	0.027
Monounsaturated fatty acids (g/1000 kcal)	12.2 ± 3.3	12.1 ± 3.1	0.994
Poly unsaturated fatty acids (g/1000 kcal)	7.2 ± 2.5	7.7 ± 2.5	0.124
Dietary fibers (g/1000 kcal)	16.2 ± 6.5	18.2 ± 7.3	0.013
Simple sugar (mg/1000 kcal)	52.6 ± 14.4	53.4 ± 14.6	0.438
Calcium (mg/1000 kcal)	538.0 ± 163.5	550.1 ± 182.5	0.263
Potassium (mg/ 1000 kcal)	1631.1 ± 417.2	1673.8 ± 471.5	0.123
Magnesium (mg/1000 kcal)	163.8 ± 32.4	179.0 ± 38.9	0.038
Sodium (mg/1000 kcal)	1994 ± 1420	1980 ± 1227	0.878

Data are presented as mean (SD) for continuous variable and number (percent) for categorical variables

MetS: metabolic syndrome; TGs: HDL-C ratio: Triglycerides: High-density lipoprotein Cholesterol ratio

The dietary intakes of the DIS components for study participants are presented in Table 2. Individuals in the highest tertile of the DIS score had lower intakes of leafy greens and cruciferous vegetables, tomatoes, apples and berries, deep yellow or orange vegetables and fruit, other fruits and real fruit juices, other vegetables, legumes, fish, high-fat dairy, low-fat dairy, coffee and tea, and nuts in compared to those in the lowest tertile. However, dietary intakes of poultry, red and organ meats, processed meats, added sugars, other fats, and refined grains and starchy vegetables were significantly increased across DIS score tertile (P < 0.05). Also, data on LIS components of study participants are expressed in Table 2. Participants in the highest tertile of LIS were significantly low active, had a higher percentage of smoking, and a higher percentage of obesity or overweight (higher BMI) compared with those in the lowest tertile of LIS.

**Table 2:** The intakes of lifestyle inflammatory score and dietary inflammatory score components in the study population

Variable	Tertiles			P-value
Tertiles of DIS median (IQR) DIS score	T1 − 4.8 (− 7.6, − 2.9)	T2 0.8 (− 0.2, 1.8)	T3 4.7 (3.6, 6.3)	
DIS components				
Leafy greens and Cruciferous vegetables (g/d)	23.4 (11.7–40.9)	13.1 (7.4–23.2)	7.1 (3.8–13.3)	< 0.001
Tomatoes (g/d)	137.2 (66.5–139.7)	69.2 (40.4–137.4)	41.0 (20.2–66.0)	< 0.001
Apples and berries (g/d)	115.6 (58.3–159.8)	53.2 (27.1–106.2)	23.0 (12.3–43.0)	< 0.001
Deep yellow or orange Vegetables and fruit (g/d)	84.2 (48.1–131.0)	38.9 (25.7–62.9)	21.2 (12.6–35.6)	< 0.001
Other fruits and real fruit juices (g/d)	347.0 (238.2–532.5)	187.2 (122.3–298.1)	113.6 (64.1–198.7)	< 0.001
Other vegetables (g/d)	173.3 (126.8–240.7)	118.8 (86.3–163.2)	81.8 (53.2–116.5)	< 0.001
Legumes (g/d)	19.5 (10.7–40.1)	13.8 (8.0–24.1)	10.3 (5.8–18.1)	< 0.001
Fish (g/d)	9.0 (4.5–16.6)	6.8 (3.7–13.1)	5.3 (2.6–9.3)	< 0.001
Poultry (g/d)	24.2 (12.1–42.5)	21.4 (12.1–36.4)	13.2 (8.5–27.0)	< 0.001
Red and organ meats (g/d)	35.3 (21.8–54.0)	30.7 (21.2–47.7)	30.2 (17.9–54.1)	0.030
Processed meats (g/d)	2.6 (0.4–6.0)	2.7 (0.7–6.1)	3.3 (1.3–7.8)	0.001
Added sugars (g/d)	58.9 (33.0–97.9)	53.8 (34.4–101.0)	50.8 (28.5–85.4)	0.193
High-fat dairy (g/d)	154.8 (65.7–242.2)	101.5 (33.4–230.0)	51.8 (17.2–121.7)	< 0.001
Low-fat dairy (g/d)	280.0 (176.7–379.0)	210.3 (116.2–324.3)	183.9 (88.2–298.3)	< 0.001
Coffee and tea (g/d)	750.0 (269.8–1000)	501.3 (251.3–753.5)	375.0 (250.0–735.0)	< 0.001
Nuts (g/d)	5.9 (2.8–12.7)	3.8 (1.9–7.5)	2.6 (1.2–4.8)	< 0.001
Other fats (g/d)	25.1 (13.3–36.8)	26.3 (13.0–37.3)	30.1 (18.9–45.0)	< 0.001
Refined grains and Starchy vegetables (g/d)	408.1 (313.7–542.5)	424.0 (333.1–554.6)	495.1 (351.8–656.5)	< 0.001

Tertiles of LIS median (IQR) LIS score	T1 − 0.18 (− 0.41, 0.01)	T2 0.71 (0.48, 0.89)	T3 1.39 (1.16, 1.57)	
LIS component				
Current smoker (%)	3.5	10.0	33.1	< 0.001
Physical activity categories (%)				0.045
Low active	28.3	30.3	36.6	
Moderately active	34.5	36.3	31.8	
High active	37.2	33.4	31.6	
BMI categories				< 0.001
Normal weight (BMI < 25) (%)	100.0	3.5	0.0	
Overweight (BMI = 25–29.9) (%)	0.0	96.5	26.1	
Obese (BMI ≥ 30) (%)	0.0	0.0	73.9	

Data are presented as mean ± standard deviation for normally distributed variables and median (25–75 interquartile range) for skewed variables

DIS: dietary inflammation scores, LIS: lifestyle inflammation scores

The OR of MetS according to tertiles of DIS and LIS is indicated in Table 3. In the age and sex-adjusted model, there was a positive association between the higher score of LIS (OR = 7.56; 95% CI 5.10–11.22, P for trend < 0.001) with the risk of MetS incident. However, the association of DIS and risk of MetS development was not statistically significant (OR = 1.30; 95% CI 0.93–1.80, P for trend = 0.127). Also, based on the multivariable-adjusted model, after controlling age, sex, BMI (for DIS), physical activity (for DIS), smoking (for DIS), educational level, daily energy intake, and baseline levels of FBS, SBP, DBP, TGs to HDL-C ratio, and waist residual BMI, the higher score of DIS (OR = 1.58; 95% CI 1.01–2.35, P for trend = 0.022) and LIS (OR = 8.38; 95% CI 5.51–12.70, P for trend < 0.001) were associated with increased the risk of 6.1-year incidence of MetS.

**Table 3:** Odds ratio (95% CI) of metabolic syndrome risk according to tertiles of inflammatory indices

	Tertiles of scores			P for trend
	Tertile 1	Tertile 2	Tertile 3	
DIS				
Median score	− 4.84 (− 7.63, − 2.93)	0.80 (− 0.27, 1.82)	4.77 (3.64, 6.31)	
Model 1^a^	1.00 (Ref)	0.96 (0.70–1.32)	1.30 (0.93–1.80)	0.127
Model 2^b^	1.00 (Ref)	1.10 (0.75–1.56)	1.59 (1.09–2.33)	0.015
Model 3^c^	1.00 (Ref)	1.11 (0.76–1.62)	1.58 (1.01–2.35)	0.022
LIS				
Median score	− 0.18 (− 0.41, 0.00)	0.71 (0.48, − 0.89)	1.39 (1.16, − 1.57)	
Model 1a	1.00 (Ref)	3.42 (2.35–4.98)	7.56 (5.10–11.22)	< 0.001
Model 2^d^	1.00 (Ref)	3.29 (2.25–4.82)	9.25 (6.07–14.09)	< 0.001
Model 3^e^	1.00 (Ref)	3.30 (2.22–4.91)	8.38 (5.51–12.70)	< 0.001

^a^Model 1: adjusted for age and sex

^b^Model 2: adjusted for model 1 and energy intakes, body mass index, smoking, physical activity, and education level

^c^Model 3: adjusted for model 1, model 2, and baseline levels of FBS, SBP, DBP, TG to HDL ratio, and Waist residual BMI

^d^Model 2: adjusted for model 1 and energy intakes and education level

^e^Model 3: adjusted for model 1, model 2, and baseline levels of FBS, SBP, DBP, TG to HDL ratio, and Waist residual BMI

The findings of the ROC curve analysis for predicting MetS incident using baseline DIS and LIS are indicated in Table 4. The accuracy of the ROC curve (AUC) for DIS was 47%, and the best cut-off value for its Z score was − 0.281, with a sensitivity of 64% and specificity of 43%. Also, AUC for LIS was 78% with the best cut-off value of 0.40 which was related to 86% and 63% of sensitivity and specificity, respectively.

**Table 4:** AUCs, optimal cut-off, sensitivity and specificity for the baseline dietary inflammatory score, and lifestyle inflammatory score in ROC analysis for predicting the incidence of MetS

	AUC (95% CI)	P	Cut-off	Sensitivity	Specificity
Dietary inflammatory score	0.47 (0.42 to 0.49)	0.045	− 0.281	0.64	0.43
Lifestyle inflammatory score	0.78 (0.69 to 0.82)	< 0.001	0.400	0.86	0.63

## Discussion

In the current study, we investigated the association of DIS and LIS and the risk of MetS in the framework of longitudinal population-based study after a 6.1-year follow-up. Findings indicated that a higher score of DIS and LIS is associated with an increased risk of MetS independent of confounding factors.

Convincing evidence suggested that systemic inflammation can play a key role in the initiation and progression of MetS [26]; in fact, the complex interaction of genetic predisposition and various environmental factors in each individual, including dietary pattern, physical activity, smoking, alcohol consumption, and metabolism is crucial in determining the levels of systemic inflammation [26, 27]. Our study indicated that a dietary pattern and lifestyle with higher pro-inflammatory characteristics may be predicting the higher risk of MetS. Although, to the best of our knowledge, there is no study on the association of inflammatory potential of diet and lifestyle determined by LIS and DIS with the risk of MetS incident; our findings are in agreement with results of some previous observational studies that have assessed the association between LIS and DIS and the risk of chronic diseases such as cancers, all-cause mortality, cancer- and cardiovascular disease-specific mortality [16–18]. Byrd et al. reported that diets and lifestyles with higher pro-inflammatory exposures, characterized by the higher score of LIS and DIS, can be related to increasing the risk of incident colorectal adenoma [18]. Also, it has been observed that pro-inflammatory diets and lifestyles are associated with a greater risk of all-cause, cancer- and cardiovascular disease-specific mortality [16]. Our findings on the association between DIS with the risk of MetS are also comparable with the results of previous studies that assessed the role of the dietary inflammatory index (DII) in the development of MetS [28]. DII as a pre-defined dietary inflammation score was developed to assess the contributions of dietary exposures on the inflammatory status and consequently the development of chronic diseases such as MetS. A meta-analysis of observational studies did not confirm that the higher score of DII was associated with the risk for MetS. In other words, the role of a dietary pattern with a high score of DII in the development the risk MetS is not yet clearly elucidate. However, we showed a direct link between the high pro-inflammatory dietary patterns (determined by DIS) with the risk of MetS. It should be noted that DIS is a novel dietary inflammatory index which can have advantages over the DII; despite the DIS, the DII mostly includes specific anti/pro-inflammatory nutrients, and may not account for the myriad other dietary components in foods, which can be responsible for inflammation. Also, the DII mostly do not consider the nutrient interactions in body homeostasis and other effects of unmeasured and unknown anti/pro-inflammatory compounds of whole foods and beverages.

Our findings suggested that participants with a higher score of DIS have higher adherence to the unhealthy dietary pattern, which is characterized by various food components that may contribute to increasing MetS development. Based on our results, this unhealthy dietary pattern is defined by a higher intake of red and processed meat, starchy food items, added sugar, and fats, and lower intakes of fruits, vegetables, nuts, dairy products, legumes, and fish. It seems that high variation in intake of the above-mentioned food groups may be associated with the risk of chronic diseases such as MetS through the effect on systemic inflammation; previous studies have reported that higher intakes of fruit and vegetable or their bioactive components are negatively related to inflammatory markers, such as C-reactive protein (CRP) and interleukin-6 (IL-6) [15]. Also, healthy dietary patterns such as the DASH diet and Mediterranean diet that emphasizes a higher intake of legumes, dairy foods, whole grain, nuts, fruit, and vegetables, and low intakes of red and processed meat and sugar, has indicated to have anti-inflammatory effects [29–31]. However, an unhealthy diet with high consumption of red and processed meats, sugar-added foods, and fast foods is associated with higher levels of inflammatory markers such as CRP and IL-6 [32]. Therefore, it is expectable, a high pro-inflammatory dietary pattern leads to an increased risk of low-grade chronic inflammation and development of MetS through-provoking in pro-inflammatory biomarkers and reduction of anti-inflammatory biomarkers [33].

In our study, the power of the LIS index (OR: 8.38) in the prediction of the increased risk of MetS was much stronger than the DIS index (OR:1.58); This large difference in the incidence of MetS was observed especially in the third tertile of LIS; based on baseline results, participants in the third tertile of LIS had a very unfavorable situation in terms of the LIS determinants including physical activity level (36.6% low active), obesity (73.9% obese) and smoking (33.1% smoked) in comparison to other tertiles of LIS. These findings were to be expected because the components of the LIS individually are very strong predictors of the risk of MetS. The cooperative contributions of major lifestyle-related factors including BMI, physical activity, and smoking to inflammation, revealed a greater association with MetS in comparison to the DIS as an alone dietary inflammatory index. Therefore, the inflammatory conditions created by these mentioned LIS determinants in participants in the third tertile of LIS can make them much more prone to increased risk of MetS. These findings have been confirmed by the results of ROC curve analysis which clearly showed that the LIS index, which includes the inflammatory effect of three important lifestyle determinants, has a higher sensitivity and specificity in predicting the risk of MetS compared to the DIS index (only indicates inflammation caused by the consumption of foods).

It is previously reported that the LIS components as lifestyle-related factors, including physical activity, BMI, and smoking may have a notable effect on the inflammatory status and metabolic homeostasis. The elevated BMI and increased adipose tissue are positively associated with inflammatory markers such as TNF-α, interleukin-6, and adipokines [26]. An increase in the plasma level of these inflammatory compounds subsequently leads to an increased risk of impaired hepatic metabolism of free fatty acids and glucose, hyperinsulinemia, insulin resistance, and dyslipidemia [12, 34–36]. Furthermore, smoking has been proposed as an independent risk factor for the development of MetS by several mechanisms including the release of nicotine, effect on adipokines levels, impaired lipid profile level, and increased inflammatory reactions [37]. In smokers, the higher inflammatory reactions can increase the risk of MetS development; because smoking has a detrimental impact on metabolism, β-cells dysfunction, and IR, which are mostly related to up-regulating inflammatory biomarkers and cytokines such as CRP [37, 38]. The higher physical activity level has protective effects on chronic inflammation via its ability to improves plasma antioxidant capacity, increasing anti-inflammatory cytokines production, reducing vascular wall inflammation [39], desirable alteration in the lipid-deposition pattern, and lowering body fat mass through negative energy balance [40]. Also, it is reported that physical inactivity is related to lifestyle‐related chronic diseases via initiation or promotion of low‐grade inflammation, indicated by higher inflammatory markers levels such as CRP, IL-6, and tumor necrosis factor‐α (TNF‐α) [41, 42]. Therefore, in view of the above, individuals with higher LID scores are mainly smokers, inactive, and high in BMI, who may be more susceptible to chronic diseases such as MetS.

The current study has several important strengths. To the best of our knowledge, this is the first study with a prospective design and long-term follow-up to investigate LIS and DIS concerning the risk of MetS. Also, valid and reliable food-frequency and physical activity questionnaires were used to assess the data on dietary intakes and physical activity levels in our study. Despite these strengths, this study has its limitations. First, although similar to epidemiological studies, in the current study valid questionnaires were used for dietary and physical activity assessment, some measurement errors are inevitable. Also, in this study, we did not have measurements of plasma insulin levels, which could have been helpful in more additional analysis and stronger interpreting the results. Finally, although major confounding variables (including age, sex, BMI, physical activity, educational level, smoking, and daily energy intake) were adjusted in our models, there may still be residual or unmeasured confounders the effects of which cannot be ruled out.

## Conclusion

The results of our population-based cohort study showed that a higher score of DIS and LIS are associated with an increased risk of MetS in adults. Therefore, our results suggest that a higher ratio of pro- to anti-inflammatory exposures can be related to increasing the risk of cardio-metabolic abnormalities.

## Data Availability

The datasets analysed in the current study are available from the corresponding author on reasonable request.

## Abbreviations

BMI: Body mass index
CIs: Confidence intervals
CVD: Cardiovascular disease
DII: Dietary inflammatory index
DIS: Dietary inflammation scores
FCT: Iranian Food Composition Table
FPS: Fasting plasma sugar
HDL-C: High-density lipoprotein cholesterol
LIS: Lifestyle inflammation scores
MAQ: Modifiable activity questionnaire
MET-h/wk: Metabolic equivalent hours per week
MetS: Metabolic syndrome
ORs: Odds ratio
TC: Total cholesterol
TLGS: Tehran Lipid and Glucose Study
TGs: Triglycerides
USDA: United States Department of Agriculture
WC: Waist circumference
